# Early intervention with *Bifidobacterium lactis* NCC2818 modulates the host-microbe interface independent of the sustained changes induced by the neonatal environment

**DOI:** 10.1038/s41598-017-05689-z

**Published:** 2017-07-13

**Authors:** Marie C. Lewis, Claire A. Merrifield, Bernard Berger, Olivier Cloarec, Swantje Duncker, Annick Mercenier, Jeremy K. Nicholson, Elaine Holmes, Mick Bailey

**Affiliations:** 1 Food and Nutritional Sciences, School of Chemistry, Food and Pharmacy, University of Reading, Reading, RG6 6AP UK; 2 Biomolecular Medicine, Department of Surgery and Cancer, Imperial College London, SW7 2AZ London, UK; 3 Nestlé Research Centre, Vers-chez-les-Blanc, 1000 Lausanne 26, Switzerland; 4 Korrigan Sciences Ltd., Maidenhead, SL6 2GN UK; 5 Infection and Immunity, Department of Clinical Veterinary Science, University of Bristol, Langford House, Langford, North Somerset BS40 5DU UK

## Abstract

Inflammatory and metabolic diseases can originate during early-life and have been correlated with shifts in intestinal microbial ecology. Here we demonstrate that minor environmental fluctuations during the early neonatal period had sustained effects on the developing porcine microbiota and host-microbe interface. These inter-replicate effects appear to originate during the first day of life, and are likely to reflect very early microbiota acquisition from the environment. We statistically link early systemic inflammation with later local increases in inflammatory cytokine (IL-17) production, which could have important enteric health implications. Immunity, intestinal barrier function, host metabolism and host-microbiota co-metabolism were further modified by *Bifidobacterium lactis* NCC2818 supplementation, although composition of the *in situ* microbiota remained unchanged. Finally, our robust model identified novel, strong correlations between urinary metabolites (eg malonate, phenylacetylglycine, alanine) and mucosal immunoglobulin (IgM) and cytokine (IL-10, IL-4) production, thus providing the possibility of the development of urinary ‘dipstick’ tests to assess non-accessible mucosal immune development and identify early precursors (biomarkers) of disease. These results have important implications for infants exposed to neonatal factors including caesarean delivery, antibiotic therapy and delayed discharge from hospital environments, which may predispose to the development of inflammatory and metabolic diseases in later life.

## Introduction

Non-communicable diseases associated with metabolism and immunity are an increasing challenge for 21^st^ century medicine. Predisposition to many of these conditions appears to originate during early-life ‘programming events’^1^. This term refers to critical points of developmental plasticity where changes in environmental factors can have long-term effects on physiological development^2, 3^. Here, ‘environmental factors’ refers to anything external to the individual including, for example, mode of birth delivery, nutritional and physiological status of the mother, siblings, nutrition and medication. One important link between such environmental factors and the development of later disease is the gut microbiota since it can be influenced by all of these factors and is a major driver for development of the host immunological^4^ and metabolic systems^5^. In laboratory animals, the pattern of early colonisation of bacterial species in the neonatal intestine influences both composition, and function of the adult microbiota^6, 7^ and we have previously demonstrated that the neonatal environment, and weaning diet, exerts a sustained influence on the development of this microbiota and metabolic phenotype^8^. This sequential colonisation pattern^9^ is difficult to predict, control or measure in human populations but once developed the adult microbiome is considered to be relatively robust with individuals categorised as one of several different enterotypes^10^. Conditions including obesity, diabetes^11^ and inflammatory bowel disease^12^ often correlate with specific, atypical microbiota composition/profiles which may originate in childhood^13^. Thus, modulation of microbial ecosystems can occur early in humans, perhaps when the composition is less stable and can be stretched beyond an ‘elastic limit’ where the changes become permanent^14^. By analogy, the changes in host metabolism and immunity resulting from this early microbial colonisation are likely to lead to long lasting imprinting. The dynamic evolution of the intestinal ecosystem in the early-life stages may represent a window of opportunity to influence the development of the microbiota, and therefore the immune and metabolic systems, through the administration of specific bacterial strains.

Although current literature links the immune system, metabolism, and the intestinal microbiota^15–19^, the molecular interactions and pathways involved in this cross-talk are complex and poorly characterized. However, the existence of these links does suggest that it is feasible to identify biomarkers in accessible biofluids, such as metabolites in urine, which may correlate with changes in less accessible sites, such as immune responses in the intestinal mucosa. Such biomarkers could be effective in the early detection of disease, monitoring disease development and for quantifying the efficacy of therapeutic intervention. There is increasing interest in identifying biomarkers of low grade inflammation which has been implicated in multiple disease processes including insulin resistance, type II diabetes and cardiovascular diseases^20–23^, as well as many gut disorders^24, 25^. Further understanding of microbiota, metabolic and immune interactions could lead to the identification of novel biomarkers of immune development, and status, and contribute to the development of stratified and personalised medicine.

Pigs are valuable comparative models for humans^26^ since they share many features of gastrointestinal physiology, immunology, metabolism, microbiology and diet^27–29^. Here we use the neonatal piglet to evaluate the effect of *Bifidobacterium lactis* NCC2818 *subsp. lactis* (*B. lactis* NCC2818) (CNCM I-3446), previously documented for its probiotic properties in human infants^30–34^, on the intestinal microbiota, metabolism and mucosal immune system when supplemented from birth. Our experimental design allowed us to identify effects of environmental variations in the immediate post-natal period on later immune, metabolic and microbial parameters. Despite considerable variation between our two experimental groups, we established that probiotic supplementation leads to robust changes in the host response that can be evidenced beyond those induced by environmental factors. Finally, we identify immune-metabolic correlations with the aim of exploring potential novel (surrogate) biomarkers in this tractable model species.

## Results

### Early *B. lactis* NCC2818 administration ameliorates inter-batch differences in intestinal barrier integrity

**Figure 1:**
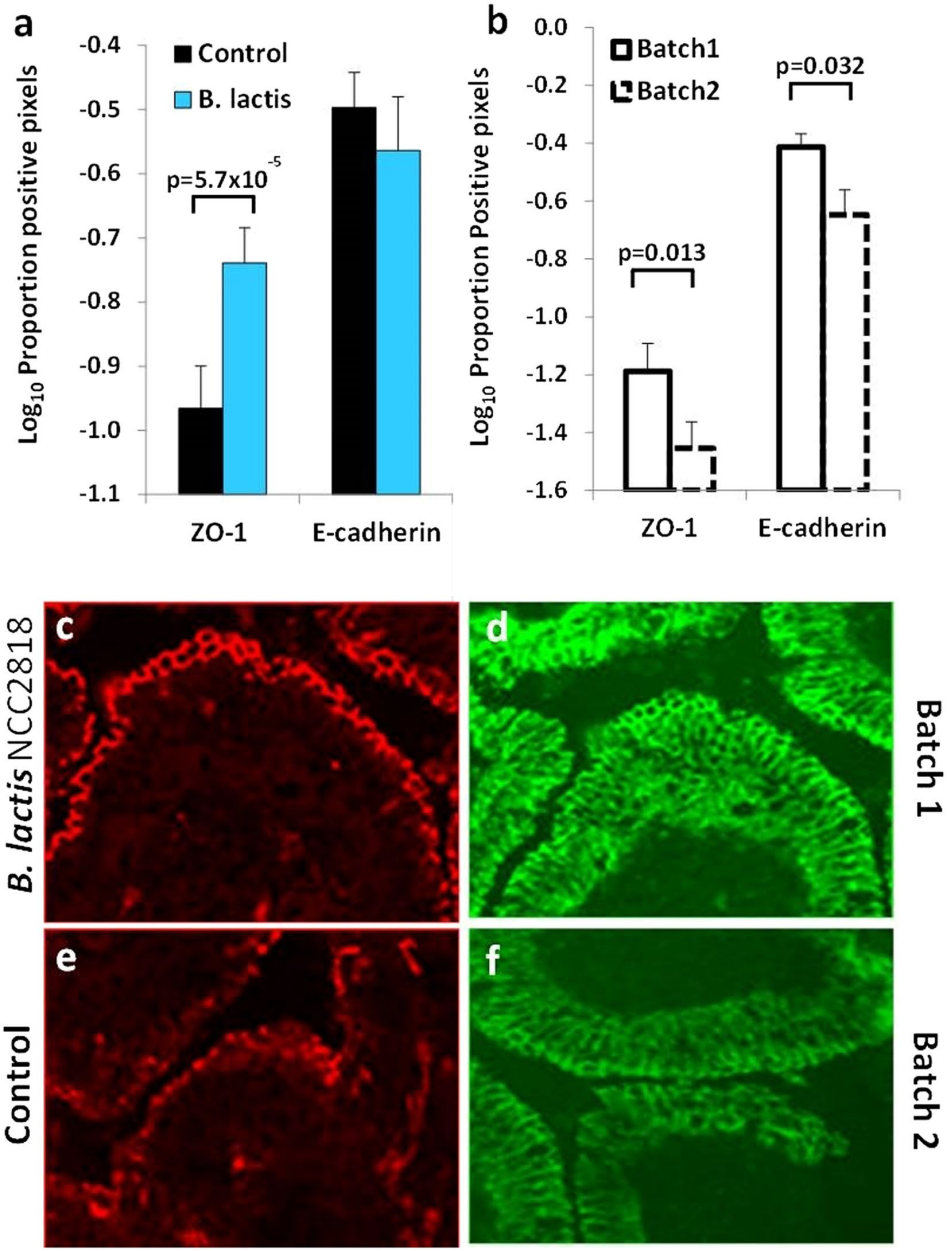
Expression of tight-cell junction proteins is increased by *B. lactis* NCC2818 supplementation and by batch. Quantitative immunohistology of ZO-1 and E-cadherin staining in jejunal epithelium in response to treatment (**a**) or related to batch (**b**). Representative images of ZO-1 staining (red) in the treatment group (**c**) and control group (**e**), and E-cadherin staining (green) in batch 1 (**d**) and batch 2 (**f**) in the villus tip. n = 6; Error Bars = SEM.

Supplementation with *B. lactis* NCC2818 was associated with increased expression of the tight-cell junction (TCJ) associated protein ZO-1 in the epithelium of the distal jejunum (*p* = 5.7 × 10^−5^, Fig. 1a, c and e). There were significant differences in expression both of ZO-1 and E-cadherin between the replicate batches, batch 1 expressing more ZO-1 and E-cadherin (p = 0.013 and p = 0.032 respectively) than batch 2 (Fig. 1b, d and f).

### Batch effects detected at 35 days old are present from 1 day after birth

**Figure 2:**
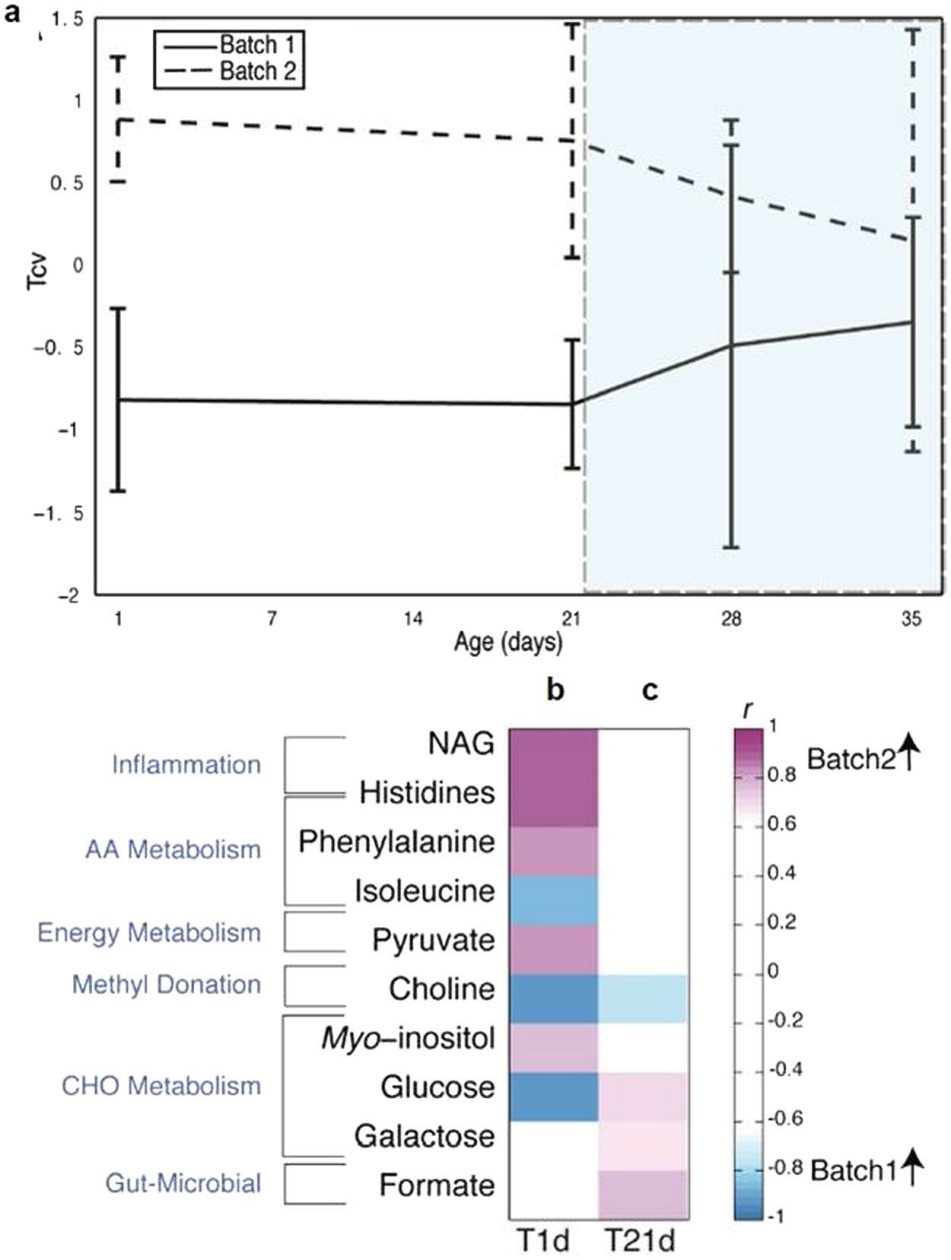
Batch effects are observed in the metabolic profile of serum at 1d. (**a**) Serum was analysed by ^1^H NMR spectroscopy and a pairwise comparison was made by O-PLS-DA (uv scaling), between the two experimental batches (n = 6) and the mean of the cross-validated scores (Tcv) from each model (+/− standard deviation) are plotted at 1, 21, 28 and 35 days. Post-weaning period depicted by blue shading. (**c**) Significant correlation coefficients (*r* > +/− 0.6) were extracted from these pairwise models and displayed as a heatmap for the model at 1d (**b**) and at 21d (**c**). Metabolites coloured in increasing shades of purple are significantly increased in batch 2 and metabolites ordered by metabolic pathway. Abbreviations: AA–amino acid; CHO–carbohydrate; NAG–*N*-acetyl glycine.

In order to determine whether batch differences arose during the feeding of probiotic or before the introduction of any experimental intervention, serum samples taken from piglets at 1 day old, before the start of the intervention, were analysed. Significant differences were observed in the serum between animals in different replicate batches from the start of the experiment (Fig. 2a). Even at 1d old, batch 1 had significantly lower levels of serum N-acetylglycoproteins (NAG), histidine, phenylalanine, pyruvate, myo-inositol and significantly higher levels of isoleucine, choline and glucose than animals in batch 2 (Fig. 2b). At 21d, batch differences were still present, although the metabolites involved had changed: the significant higher level in serum choline in batch 1 at 1d was conserved, while glucose, galactose and formate levels were lower in batch 1 but higher in batch 2 (Fig. 2c). Differences in serum metabolites between batches were reduced after weaning (21d), and at the end of the experiment (35d), there were no significant differences observed in serum between the two batches. In contrast, no significant effect of *B. lactis* NCC2818 supplementation on the metabolic profile of serum was observed at any point during the experiment (data not shown).

### Mucosal cytokines synthesis is affected by both experimental batch and early supplementation with *B. lactis* NCC2818 while early inflammatory markers predict later cytokine synthesis

**Figure 3:**
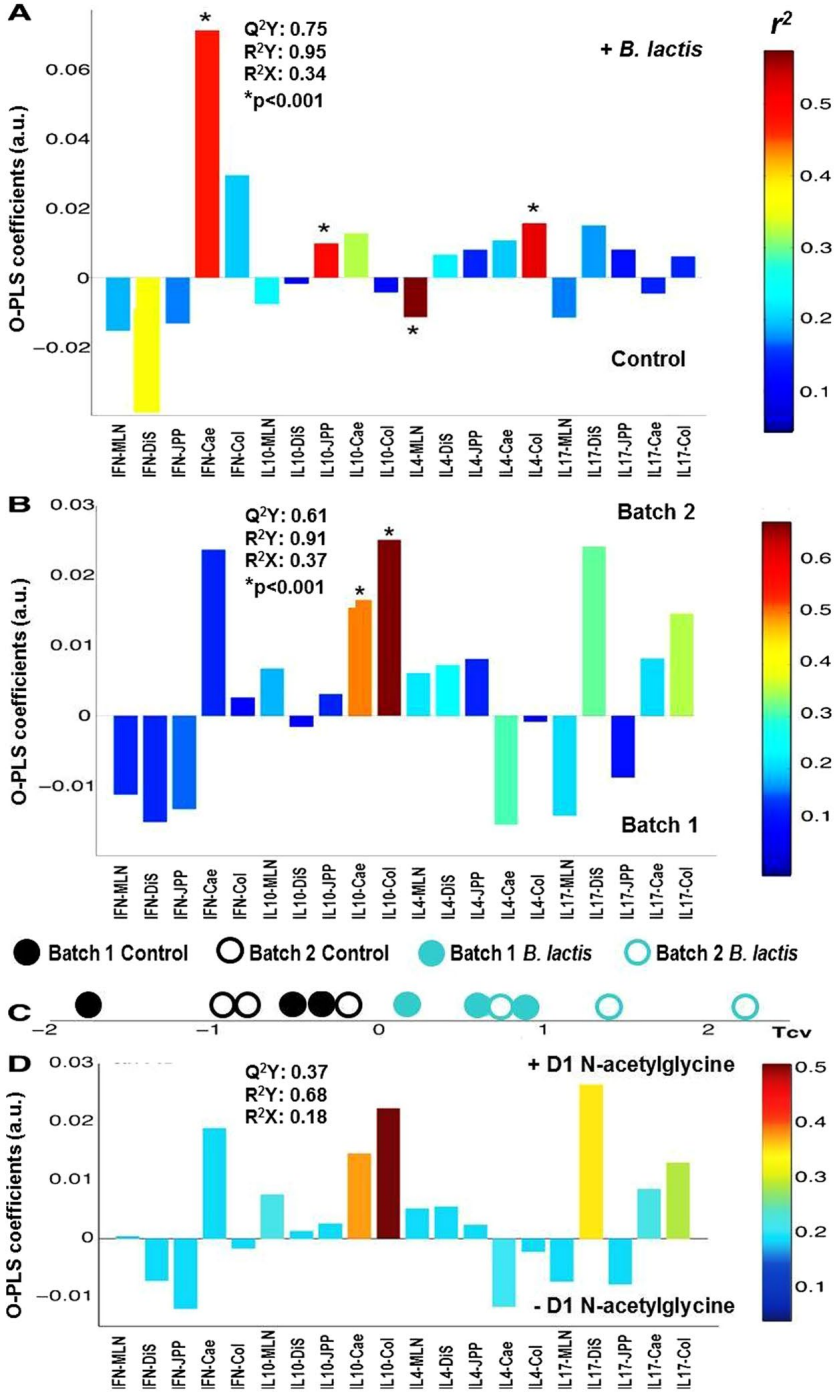
Mucosal cytokines synthesis is affected by both experimental batch and early supplementation with *B.lactis* NCC2818 while early inflammatory markers predict later cytokine synthesis. Back-scaled correlation covariance plots derived from a pairwise O-PLS-DA model comparing the effect of *B. lactis* NCC2818 supplementation on cytokine production in immunological tissues (**a**). The bars pointing upwards denote cytokines that are relatively increased in animals supplemented with *B.lactis* NCC2818 from 1 day (n = 6)–an increasing colour intensity of red depicts the strength of this association (*r* ^*2*^) and model statistics can be seen in the upper left hand corner. The experimental batch significantly affected 35 day cytokine production (**b**); the bars pointing upwards denote those cytokines that are relatively increased in batch 2 (n = 6). The cross-validated scores of O-PLS model using 24-hour serum N-acetylglycoproteins as a Y variable to predict 35 day cytokine production is shown in (**c**) with the legend above. Figure (**d**) depicts the corresponding loadings plots with bars above the axis representing cytokines associated with a relatively higher level in 1 day serum NAG.

Functionally, supplementation with *B. lactis* NCC2818 was linked to a significant decrease in IL-4 production from MLN, and significantly increased production of IL-4 from colon, interferon gamma (IFNγ) from caecum and IL-10 from the JPP (Fig. 3a), Effects associated with probiotic supplementation were detected despite the presence of underlying differences in the production of these proteins between the two experimental batches: piglets in batch 2 had significantly increased IL-10 secretion from both the caecum and colon compared to batch 1 counterparts (Fig. 3b). The unprocessed cytokine data are available as Supplementary Fig. 1.

The observation that markers of an acute phase response (NAG) were relatively increased in the animals in batch 2 at 1 day old, coupled with the increased regulatory cytokine expression in the large intestine at 35 days old in the same animals led us to postulate if early inflammatory response could predict later cytokine production. An O-PLS model was performed using the relative concentration of NAG as the Y matrix and the 35 day cytokine profile as the X-matrix. The results (Fig. 3d) demonstrate a statistical correlation between increasing levels of NAG at 1 day and increasing levels of colonic and caecal IL-10 and distal small intestinal IL-17 at 35 days. The distribution of animals from the different batches in the scores plot (Fig. 3c) from this model indicates that this is not simply an artefact of batch. In fact, animals supplemented with *B.lactis* NCC2818 induced a more pronounced tolerogenic cytokine response linked to increased 1 day NAG.

### Supplementation with *B. lactis* NCC2818 reduced inter-batch variation in mucosal antigen expression

**Figure 4:**
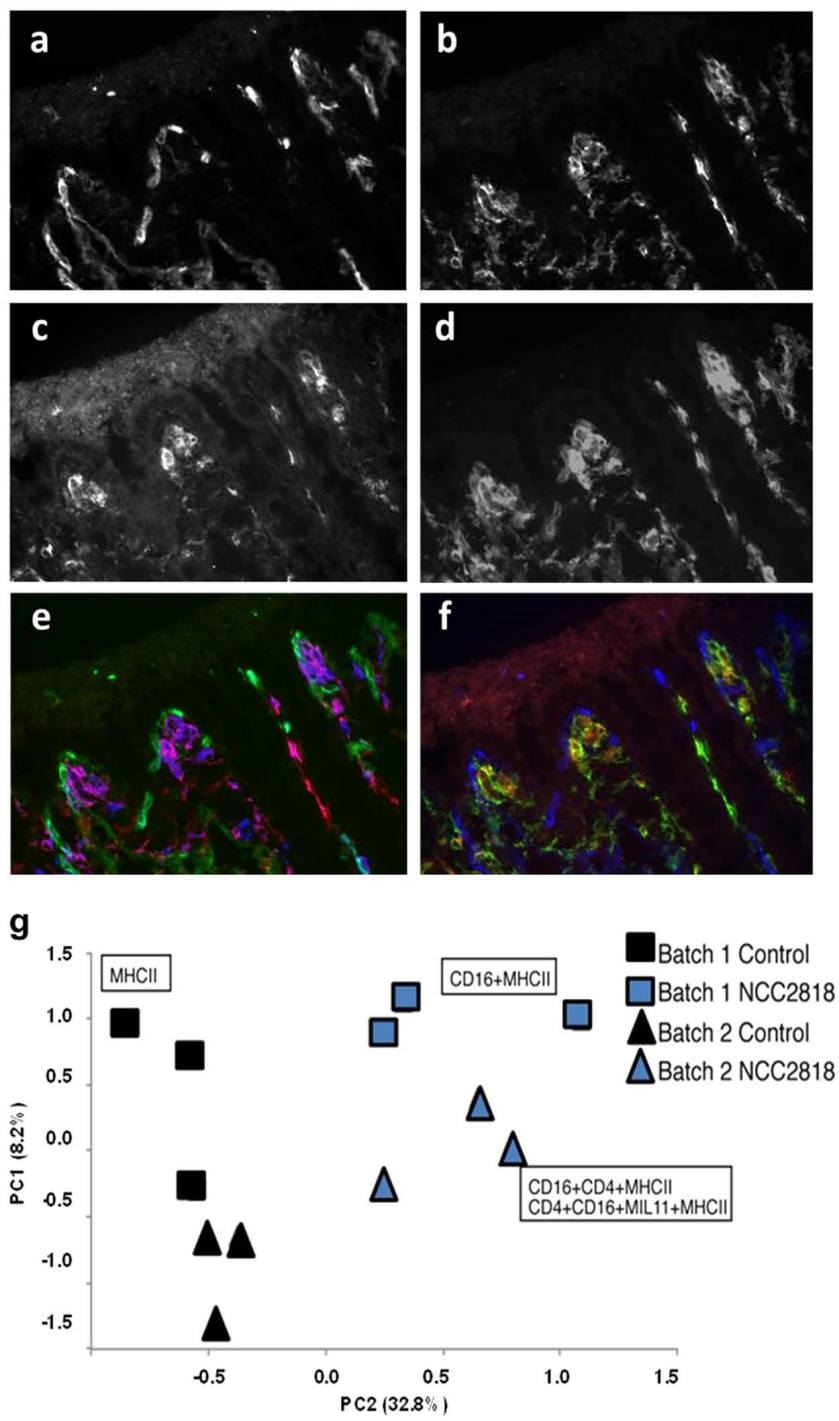
Reduced inter-batch variation in mucosal antigen expression in animals supplemented with *B.lactis* NCC1818. Representational images of CD4 (**a**), CD16 (bone-marrow-derived APC) (**b**), MIL11 (intestinal epithelium) (**c**) and MHCIIDR (**d**) staining in colonic tissue of a control piglet. Representational image of co-staining with CD16 (red), MIL11 (green) and MHCIIDR (blue) (**e**) and co-staining with CD4 (red), CD16 (green) and MIL11 (blue) (**f**). Co-localisation of MHC class II with, endothelial cells (MIL11) and CD4^+^ T-cells determined by principal component analysis. Principal component 1 was not associated with either treatment (n = 6) or batch (n = 6). Principal components 2 and 3 for each piglet, identified by treatment (colour) and batch (symbol).

Principle Component Analysis (PCA) of quantitative immunohistology identified two components accounting for more than 40% of the variance in expression and co-localisation of CD4, CD16, MHCII and the MIL11 epitope (gut capillary endothelium) in colonic mucosa. Both supplementation with *B. lactis* NCC2818 and experimental batch were associated with clustering in the PCA scores (Fig. 4g). Examination of the loadings associated with these two components showed that supplementation with *B. lactis* NCC2818 was linked to differences in the presence of professional antigen-presenting cells (co-expression of CD16 and MHCII), presentation by these cells to CD4^+^ T-cells (co-expression of CD16, MHCII and CD4) and recruitment of endothelial cells to this interaction (CD16, MHCII, CD4, MIL11). Additionally, supplementation with *B. lactis* NCC2818 resulted in less inter-batch variation between animals than in the control group, where the separation of animals due to batch was more apparent. The unprocessed antigen presentation data are available as Supplementary Fig. 2.

### Probiotic administration and experimental batch influence gut-microbial co-metabolism

**Figure 5:**
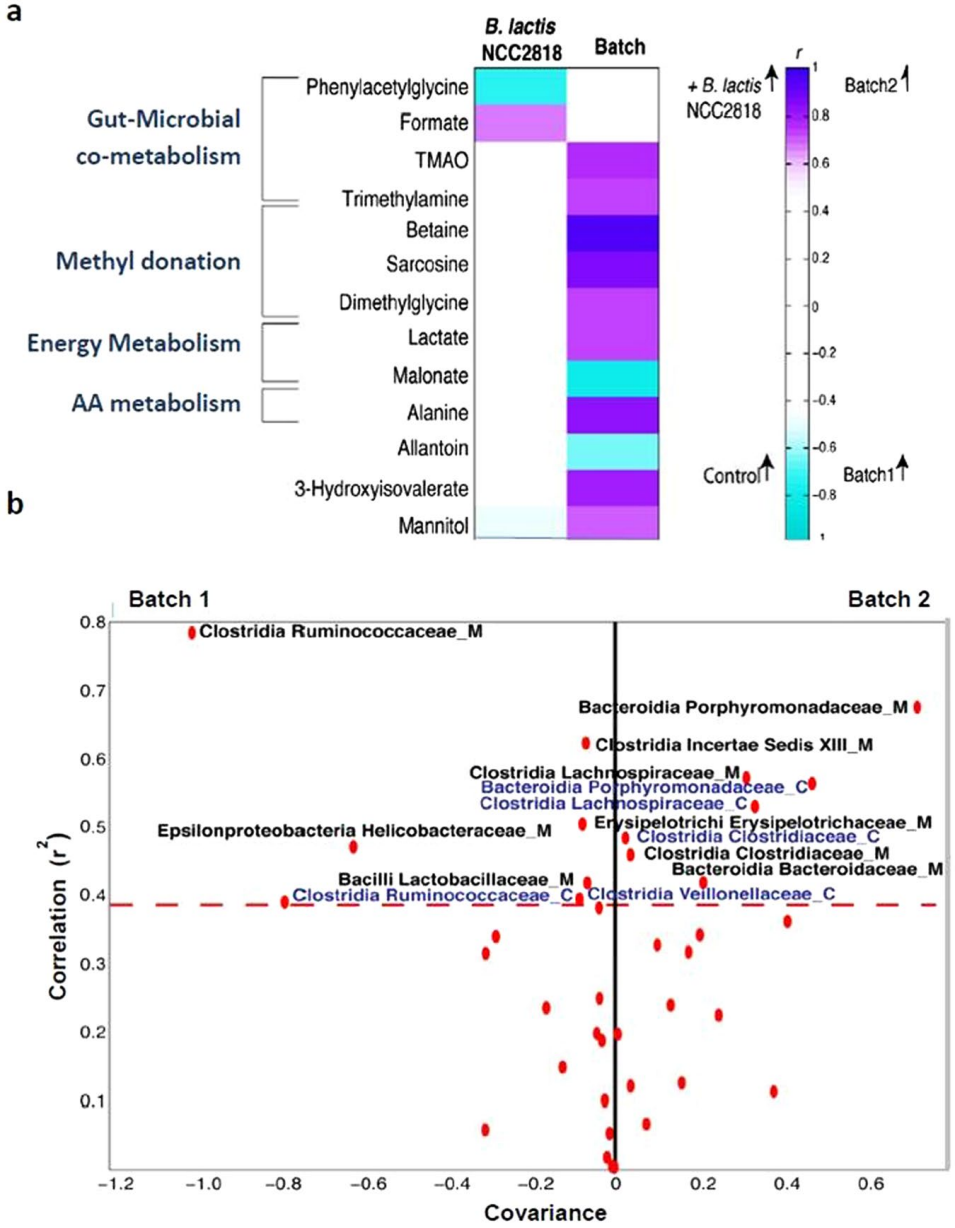
Probiotic administration and experimental batch influence gut-microbial co-metabolism, but only batch is associated with changes in the composition of the microbiota. (a) Correlation coefficient heatmap derived from 2 pairwise O-PLS-DA models comparing the effect of *B. lactis* NCC2818 supplementation (n = 6) to controls (n = 6) and comparing experimental batches (n = 6). Metabolites are ordered by their biological similarity and those coloured in increasing shades of purple are significantly increased in batch 2 or after *B. lactis* NCC2818 supplementation, while those in increasing shades of blue are relatively increased in batch 1 or the control group. (b) Volcano plot derived from a pairwise O-PLS-DA model comparing bacteria at family level between batches. Suffix ‘_C’ denotes colonic content and ‘_M’ colonic mucosa; the red dots mark the coordinates of each family’s correlation (*r* ^2^) and covariance. The points in the upper left quadrant denote those bacterial families that are increased in batch 1 whereas those points present in the upper right quadrant are relatively increased in batch 2. Those points above the red dashed line (at 0.4) are considered to be above the significance threshold. Abbreviations: AA–amino acid; TMAO–Trimethylamine-*N-*oxide.

Dietary supplementation with *B. lactis* NCC2818 resulted in a significant decrease in urinary excretion of phenylacetylglycine (PAG), while formate excretion increased (Fig. 5a). Experimental batch significantly influenced the urinary metabolic profile at 35d: urinary excretion of metabolites involved in methyl donation and choline metabolism (trimethylamine, betaine, sarcosine, dimethylglycine) were significantly higher in batch 2 than in batch 1, as were TMAO, alanine and 3-hydroxyisovalerate. In addition, urinary malonate and allantoin were significantly higher in batch 1 compared to batch 2. Urinary mannitol excretion was the only metabolite affected by both batch and probiotic intervention, being higher in batch 2 and decreased by supplementation with *B. lactis* NCC2818 (Fig. 5a).

Interestingly, there were no significant effects of *B. lactis* NCC2818 supplementation on the composition of the colonic microbiota at 35d, either in the luminal contents or the mucosa (data not shown). However, significant differences in the colonic bacterial populations were observed between experimental batches (in both treated and control groups), particularly in relation to *Clostridia* and *Bacteroidia* (Fig. 5b). The most pronounced differences between the two batches were reproducibly observed with both luminal and mucosal microbiota, strongly raising confidence in this observation.

### Urinary metabolite concentrations are significantly correlated to immunological parameters

**Figure 6:**
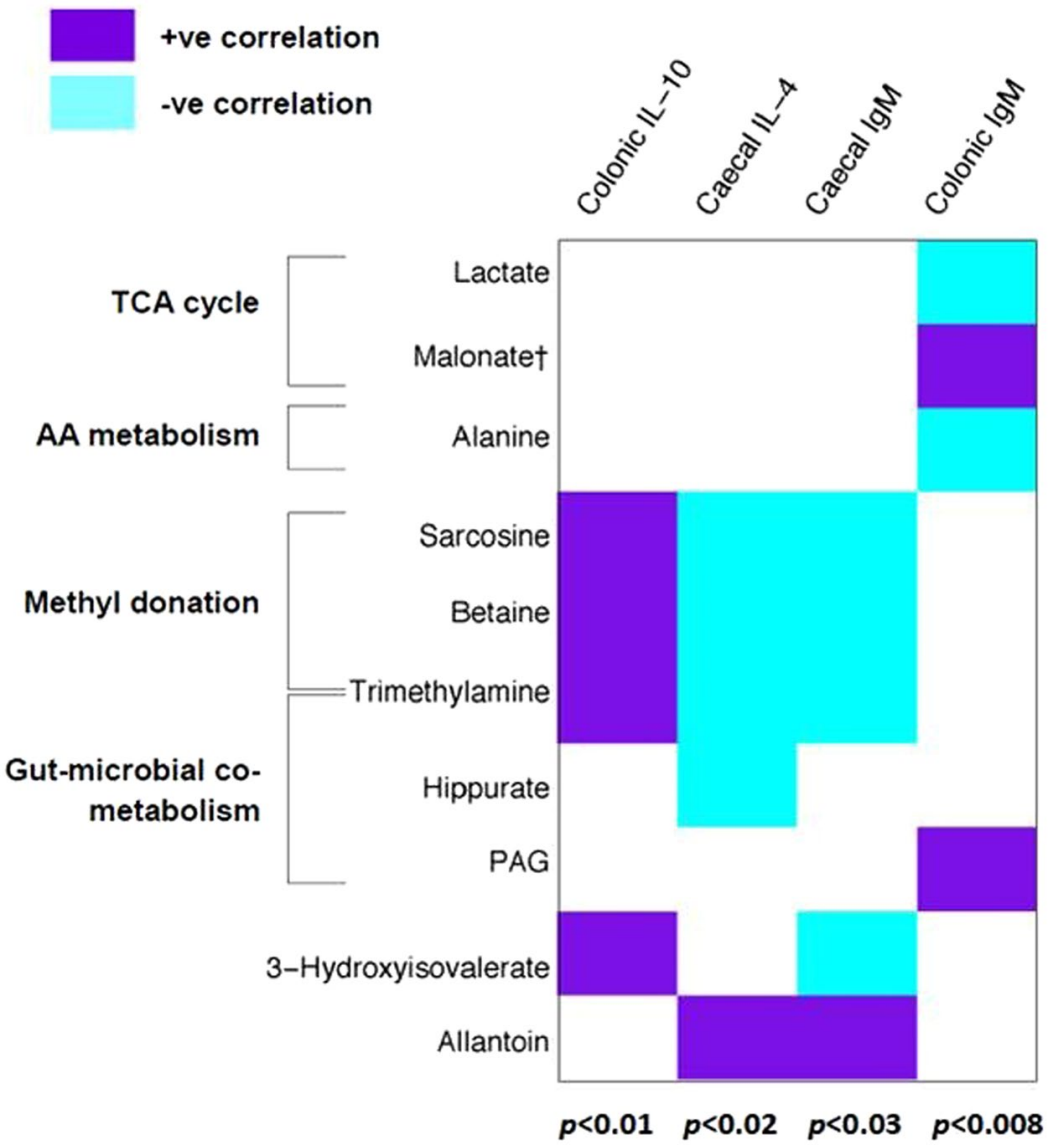
Urinary metabolite concentrations are significantly correlated to immunological parameters. Heatmap derived from 4 separate O-PLS models linking the urinary metabolic profile to immunological parameters (n = 12). Metabolites are grouped by biological similarity. ^†^Denotes a tentative assignment, PAG–phenylacetylglycine, AA–amino acid, TCA–tricarboxylic acid. Writing in bold to the left denotes putative metabolic pathway involvement (n = 12).

Immunoglobulin and cytokine parameters from all piglets, irrespective of batch and whether they received *B. lactis* NCC2818 supplementation, were compared to urinary metabolic profile and a number of significant correlations were identified across this immuno-metabolic interface (Fig. 6). These included positive correlations between colonic IL-10 synthesis and urinary metabolites associated with methyl donation (sarcosine, betaine and trimethylamine) (p = 0.01), and between malonate (associated with the TCA cycle) and colonic IgM (p = 0.008). Interestingly, this analysis also identified significant negative correlations between methyl donation-associated metabolites and caecal IL-4 (p = 0.02) and IgM (p = 0.03). Further, metabolites linked to the intestinal microbiota were significantly correlated with immune parameters, consistent with the microbiota influencing immune development: hippurate was negatively correlated with caecal IL-4 (p = 0.02) while PAG was positively correlated with colonic IgM (p = 0.008). The unprocessed IgM data are available as Supplementary Fig. 3.

## Discussion

Supplementation with *B. lactis* NCC2818 from the first day of life had significant effects on intestinal barrier function, development of the mucosal immune system and metabolic function in these outbred piglets. Batch effects are a consistent occurrence in animal studies and reflect the inconsistencies observed within free-living human populations. The observation that the overall effects of probiotic supplementation appear robustly sustained across all domains, even in the presence of a significant, additional effect of experimental batch, is an encouraging finding.

The influence of supplementation with *B. lactis* NCC2818 on intestinal barrier function was apparent in two separate platforms. The cytoplasmic protein ZO-1 associates with claudins in the tight-cell junctions, contributing to epithelial barrier integrity. Mannitol is not produced endogenously or by the microbiota, and is normally too large to cross the intestinal epithelial barrier. Its appearance in urine therefore is associated with transfer of dietary mannitol across an intestinal epithelium with decreased integrity, as occurs around weaning. Supplementation with *B. lactis* NCC2818 increased expression of epithelial ZO-1 and decreased excretion of urinary mannitol even in the presence of differential mucosal barrier integrity between replicates. Reduced epithelial barrier integrity is associated with the development of intestinal inflammatory conditions, promotion of translocation of opportunistic pathogens into the portal venous system and may compromise mucosal healing. However, the link between intestinal permeability and health has been comprehensively reviewed elsewhere^35^. In humans, administration of *B. lactis* NCC2818 to newborn infants was shown to promote immune development^31^ and response to vaccination^32^ whilst in preterm infants, *B. lactis* NCC2818 was shown to decrease intestinal permeability^36^. Here we provide further evidence that *B. lactis* NCC2818 is able to reinforce intestinal barrier integrity in the first days of life irrespective of the starting status.

Although *B. lactis* NCC2818 had no discernible effects on the composition of the microbiota at the family level, it did affect the urinary host-microbial metabolic profile (e.g. increased phenylacetylglycine and decreased formate), indicating that supplementation influenced the metabolic function of the microbiota, rather than the composition, at least at this taxonomic resolution and sequencing depth. The host and microbiome communicate via several distinct axes and the alteration in host-microbial metabolism we observed could have occurred through direct interactions with the microbiota or, indirectly, through modulation of the immune system. Consistent with the latter, supplementation with *B. lactis* NCC2818 resulted in changes in multiple immunological read-outs, including local production of immunoglobulins and cytokines in multiple tissues, and expression of antigen-presenting cell function in colonic mucosa. However, the results do not clearly indicate preferential effects on specific immunological pathways: in different tissues, increases were seen in the Th1 cytokine IFN-γ, the Th2 cytokine IL-4 and the regulatory cytokine IL-10. Nevertheless, supplementation with *B. lactis* NCC2818 was shown to reduce the differences in antigen presentation observed between the two batches.

Batch effects are a consistent occurrence with animal studies and are often attributed to differences in the environment in which the study takes place. However, such batch effects are likely to represent the kind of variation seen in the human populations which are the target of the interventions developed in animal trials. Understanding the sources of variation other than genetic background could improve the success rate of translation from animal trials to humans. In the experiments here, it was possible to identify some of the sources of batch variation. Since the environments in which the individually housed piglets were maintained from day 2 were highly controlled, and since the batch differences were observed prior to entry into this environment, it is unlikely that the variation developed during the course of the nutritional intervention. This is further supported by the observation that differences between batches in serum metabolic profile on entry into the trial disappeared after 21 days, indicating convergence, although urine profiles were still different at 35 days. It seems more likely that the source of the observed batch effects was differences in the environments of the piglets either *in utero* or, since the sows were co-housed and each batch was derived from several sows, more likely in the first day after birth. Intriguingly, some of these differences were associated with inflammation (NAG, and histidine) although no observable health differences were identified between the batches either on the farm (maternal or piglet) or later in the isolator (piglet) and all animals survived and furthermore appeared healthy throughout. Serum from piglets in batch 2 contained significantly higher levels of these inflammation-associated metabolites and, although we cannot be sure of intestinal permeability at this time point, later measurements of epithelial cell-cell adhesion molecules demonstrated reduced intestinal barrier function in these piglets and it is possible that these two findings are linked. Inflammation is most often studied in adulthood, but there is growing evidence that the early life environment has long-term, independent effects on immune regulation^37^ and inflammation, and is linked to exaggerated inflammatory responses to *in vitro* bacterial challenge^38, 39^. Importantly in this study, the animals which had a more ‘inflammatory’ phenotype at day 1 went on to secrete significantly increased levels of jejunal IL-17 at 35 days (and to a lesser extent in colonic tissue), despite being reared in the same high-hygiene environment. Increased expression of Il-17-producing intestinal-specific Th17 phenotypes have been causally linked to several inflammatory gut disorders^40, 41^ and are being progressively implicated in the gut-brain axis and ill health outside the gut, including autoimmune neuroinflammation^42^. However these animals also secreted more of the regulatory cytokine IL-10 at 35 days in large intestine sites (caecum and colon) indicating further long-term immunomodulation driven by early life events. Both batch and *B. lactis* NCC2818 supplementation had distinct effects on host microbial-metabolic interaction, with urine and serum providing a more focused window of effect at different time points. We have observed previously^43^ that serum contains fewer microbial derived compounds in comparison to urine and that it is under tighter homeostatic control than urine, which is strongly influenced by environmental factors. This current study reinforces the idea that subtle environmental variation is far more likely to be detected by analysing the urine than the serum in the context of the microbiome and nutrition.

While both batch and treatment effects were associated with alterations in host-microbial metabolism, only batch was linked directly to modifications in the composition of the gut microbiome, suggesting that the effect observed here was linked to initial microbial colonisation during the first day of life. The differences in the microbiome identified between the batches were apparent even at the family level. There was a significantly larger population of members of the *Ruminococcaceae* family in batch 1 piglets compared to batch 2. Members of this family are known to reside in the mucosal folds of the mammalian colon and populations of microbes in this location remain largely uncharacterized^44^. However, several studies have linked reduced intestinal colonisation by members of the *Ruminococcaceae* family to chronic intestinal disorders such as IBD in humans^45–47^. Microbiome analysis of intestinal mucosal scrapings also showed that batch 2 piglets harboured higher levels of *Bacteroidia Porphyromonadaceae* family members. While the direction of causality is unclear, it has been shown that *Porphyromonas* were enriched in human colorectal tumours compared to healthy controls and may be implicated in increased risk of disease development^48^. It is important to note that these families constitute normal commensal intestinal bacteria and their presence or absence is not a biomarker of disease, but instead the link is to their relative abundance, and possibly their activity.

In summary, our results demonstrate that supplementation with *B. lactis* NCC2818 from 1 day old improved intestinal barrier function and had significant effects on the development of the mucosal immune system and metabolic function. However, these effects were superimposed on a background of sustained batch differences. The experimental design, and the observed differences at one day, indicate that these batch effects are most likely attributable to differences in acquisition of intestinal microbiota immediately after birth, and that these differences may then determine subsequent development of metabolic and immunological function. Consequently, we suggest that the early life environment, and development of the early microbiota, should be taken into account during the design and interpretation of animal trials. Furthermore, understanding the causes of batch variation may contribute to improving translation from animal models to human populations. Nevertheless, the physiological effects of very early supplementation with *B. lactis* NCC2818 were consistent across both batches and therefore, in the longer term, could offer the potential to improve outcomes for ‘at-risk’ infants which have undergone early-life events linked to aberrant patterns of intestinal colonisation, for example caesarean birth or antibiotic therapy. Finally, irrespective of batch and probiotic treatment, robust links between mucosal immunological parameters and the urinary metabolic profile are demonstrated by the correlations we observed in this model: these associations raise the possibility that accessible metabolic profiling may provide valuable biomarkers to assess previously inaccessible parameters including the function of the microbiota and associated intestinal immune system in future clinical trials.

## Materials and Methods

### Animals

**Figure 7:**
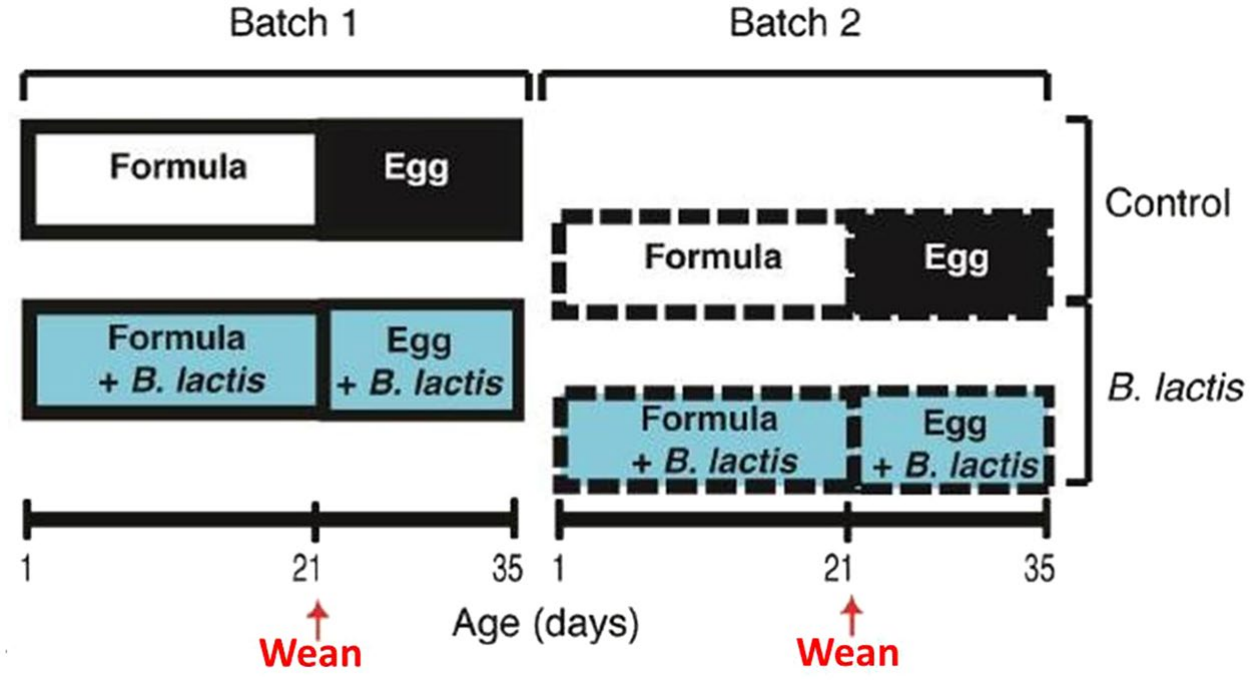
Animal trial: Schematic representation of the experimental setup depicts batch 1 (n = 6), batch 2 (n = 6), treatment (supplemented with *B. lactis* NCC2818 *from* 1 day old, n = 6) and untreated groups (no *B. lactis* NCC2818, n = 6). Piglets were fed formula milk before being weaned at 21 days (denoted by red arrow) onto an egg protein-based diet until the experiment concluded at 35 days. A detailed schematic of the experimental design is available in the supplementary data (Supplementary Fig. 1).

Animal study was conducted at the School of Clinical Veterinary Sciences (University of Bristol) and performed according to ethical guidelines under a UK Home Office License, approved by the University of Bristol ethical review group. Six healthy, large white hybrid, out-bred sows (2–3 parity) were chosen randomly from a commercial herd of 250, all housed in a single barn. Three sows (batch 1) were artificially inseminated using semen from 1 boar and 8 weeks later 3 additional sows (batch 2) were artificially inseminated using semen from the same boar. Shortly before giving birth, batch 1 sows were relocated to the farrowing unit and 2 healthy piglets from each of the resulting litters (n = 6) were taken to an isolator facility at 1 day old after receiving standard husbandry iron via an IP injection, but no antibiotics. This highly controlled, standardised environment (SPF, HEPA filtered, positive pressure) was thoroughly cleaned and fumigated with formaldehyde gas (Alphagen Prills pellets, Antec AH Int., Sudbury, UK) before each experiment. The piglets were housed together within this isolator for 1 day while they learnt to drink a pig adapted bovine milk-based formula (Piggimilk^®^,Volac Ltd, Lincolnshire, UK, supplementary table 1), similar to that given to human infants (with or without probiotics accordingly). At 2d, the piglets were moved to individual units within the isolator before being sibling- and gender-matched into two groups at weaning (21d). The piglets were weaned onto an egg-based solid (slurry) diet, which was nutritionally balanced and supplemented to meet their requirements (Volac Ltd, Sleaford, Lincolnshire, UK, supplementary table 1). One group received *B. lactis* NCC2818 intervention in the form of spray-dried culture mixed into the formula and weaning feed at a concentration of 4.2 × 10^6^ colony forming units (CFU)/g, equivalent to 2 × 10^9^ CFU/kg metabolic wt/day). The remaining group (control group) received identical diets (same batch of feed), but without the probiotic and a biosecurity barrier was established to avoid crossover between the two groups. Four weeks after batch 1 sows vacated the farrowing unit, batch 2 sows entered the same unit in preparation for birth. Again, 2 healthy piglets were removed from each of the 3 batch 2 sows at 1 day old and underwent exactly the same process as piglets in batch 1. This experimental design (Fig. 7) resulted in n = 6, gender-matched, litter-matched piglets in each treatment group (Control and probiotic) and n = 6 gender-matched piglets in each replicate (batch 1 and batch 2). The experimental design is detailed in Supplementary Figure 4. The number of piglets used was based on our previous experiments^37, 43, 49–53^ where using litter as a factor during the statistical analysis reduces the number of animals required.

All piglets were bled by venipuncture at 1d (immediately following removal from the mothers), 21d (immediately before weaning) and 28d old and samples stored at −80 °C. At 35d, piglets were euthanised with an overdose of pentobarbitone (Euthatal^®^, Merial Animal Health, Essex, UK). At post-mortem, heart blood was obtained and serum was snap-frozen, along with urine aspirated directly from the bladder, in preparation for metabonomic profiling. Colonic (descending; adjacent to the colo-rectal junction) contents and mucosa were removed and snap-frozen for 16S pyrosequencing. Mesenteric lymph node (MLN), jejunal Peyer’s patch (PPs), proximal and distal jejunum (not containing PPs), splenic tip, caecum tip and the ascending portion of the spiral colon were removed for organ fragment culture (OFC). Additional colonic and distal jejunum samples from the same locations were mounted in OCT embedding matrix, snap frozen and then stored at −80 °C for later fluorescence immunohistological analysis.

### Sample Preparation for ^1^H NMR

#### Serum

Blood serum samples were prepared by the addition of 350 µl 0.9% saline solution containing 10% D_2_O (to act as a spectrometer field frequency lock) to 200 µl of serum. This mix was then vortexed, centrifuged at 12,000 *g* for 20 minutes and transferred into 5 mm outer diameter NMR tubes in preparation for analysis.

#### Urine

Urine samples were prepared by the addition of 220 µl of a 1 mM 3-trimethylsilyl-1-[2,2,3,3,^−2^H_4_] proprionate (TSP), 3 mM sodium azide (NaN_3_) and 80/20 (v/v) H_2_O/D_2_O phosphate buffer solution (pH 7.4) to 440 µl of urine. This mixture was vortexed, left to stand for 20 minutes and then centrifuged at 12,000 *g* for 20 minutes. Six hundred µl of the supernatant was transferred to 5 mm outer diameter NMR tubes^54^.

### ^1^H NMR Spectroscopy

#### Serum

Serum samples were collected longitudinally throughout the experiment as previously described. The ^1^H NMR metabolic profiles of these samples were acquired on an Avance 600 MHz NMR spectrometer (Bruker Biospin) and spectra were collected using a BACS autosampler with a Bruker 5 mm TXI triple resonance probe at 300 K. 256 scans (16 dummy scans) were collected into 64 k data points over a 20 ppm spectral width using a Carr-Purcell-Meiboom-Gill (CPMG) spin echo sequence, incorporating an echo time of 200μs to highlight contributions from low-molecular weight moieties.

#### Urine

The metabolic profile of urine from each of the piglets (collected at 35 days) was obtained by NMR spectroscopy. All spectra were acquired on an Avance 600 MHz NMR spectrometer (Bruker Biospin) and spectra were collected using a BACS autosampler with a Bruker 5 mm TXI triple resonance probe at 300 K. 256 scans (16 dummy scans) were collected into 64 k data points over a 20 ppm spectral width using the first increment of a standard one dimensional experiment with water presaturation during the relaxation delay of 2 s and during the mixing time of 100 ms.

### Metabolite Identification

Metabolites were identified by further 2D NMR experiments (*J*-RES, TOCSY, HSQC) as described previously^43^ for structural assignment, by using extant literature^43, 55^ and by using databases such as the Human Metabolome Data Base (HMDB): http://hmdb.ca/ ^56, 57^ and the Biological Magnetic Resonance Data Bank (BMRB) http://www.bmrb.wisc.edu/, along with SBase/Amix (Bruker BioSpin 2006) and NMR Suite (Chenomx, Edmonton, Canada).

### Quantification of the gastrointestinal microbiota using 16 S DNA pyrosequencing

DNA was extracted from faecal and colonic content samples using the Qiagen EZ1 kit (Qiagen, Germany) after mechanical disruption as follows: for colon content, 100 mg of material was placed in lysozyme buffer (50 mg/ml) with 0.3 g of glass beads, disrupted in a bead-beater at maximal speed for 1 min and then incubated at 37 °C for 15 min; for colon tissue, 200 mg were placed in Lysing Matrix Tubes D with lysozyme buffer and homogenised with FastPrep-24 (MP Biomedicals, Solon, OH 44139), 2 × 20 sec speed 6, with 5 min break. After centrifugation (5 min, 200 g), supernatant was transferred into tubes with 0.3 g of glass beads, disrupted in a bead-beater at 4 °C at maximal speed for 1 min and then incubated at 37 °C for 15 min.

The V1, V2 and V4 regions of the 16 S genes contained in the DNA extracts were amplified, sequenced in multiplex using the GS FLX System (Roche, Switzerland), and analyzed as previously described^58^, except for the confidence threshold of the RDP Classifier which was set to 60%, as in previous published studies^59, 60^. All data analysis was conducted on the mean relative proportion of bacteria (%). Due to the high proportion (~35%) of unclassified bacteria of pig origin identified at the genus level, we have used the family level as a trade-off between the coverage of the microbial population and the depth of taxonomical analysis.

### Organ Fragment Culture

As previously described^61^, 4 cm^2^ of the intestinal samples and 1 cm^3^ of MLN and spleen were vigorously washed in Ca^2+^ and Mg^2+^-free Dulbecco’s PBS (Sigma) containing 0.5 μM EDTA (Sigma), 1 M HEPES (Invitrogen, Paisley, UK) and 50 μg/ml gentamycin (Gibco, Paisley, UK), followed by 3 further washes in Dulbecco’s PBS containing 1% HEPES and 50 μg/ml gentamycin before being placed in RPMI1640 (Sigma, Gillingham. UK) containing 10% FCS (PAA, UK), 200 mM L-Glutamine (Invitrogen, Paisley, UK), 20U/ml streptomycin/penicillin (Invitrogen) and 50 μg/ml gentamycin (complete medium). Intestinal tissues were cut into 3 mm^2^ fragments while spleen and MLN were cut into 2 mm^3^ fragments and one fragment was placed in each of 6 individual wells (Corning Incorporated, UK) containing 1 ml of complete medium. Cultures were incubated at 37 °C, 5% CO_2_, 100% humidity for 96 h, after which they were frozen at −20 °C. The plates were thawed and the spent medium from each of the 6 duplicate wells was pooled and refrozen for later analysis.

### Cytokine production in organ fragment cultures

Supernatants from OFCs were analysed by ELISA for cytokine production using Swine IFNγ, IL-4 and IL-10 Cytoset kits (Biosource, Paisley, UK) and interleukin-17 was detected using the Swine IL-17 VetSet (Kingfisher Biotech, Saint Paul, Minnesota, US). All kits were used in accordance with the manufacturer’s protocols. Absorbance was read at 450 and 650 nm and concentrations of cytokine were determined by interpolation of samples onto the standards.

### Total IgM production in organ fragment cultures

Catching ELISA was carried out to determine total IgM in spent medium from OFCs. Briefly, 96 well microplates were coated with affinity purified goat anti-pig IgM (Bethyl Laboratories, Montgomery, Texas, USA). Serial dilutions of serum samples and reference standard were added to coated plates and incubated for 2 h at room temperature. Bound immunoglobulins were detected using isotype-specific monoclonal antibodies (anti-pig IgM K52.1C3, from Serotec) followed by HRP-conjugated goat-anti-mouse IgG_1_. Concentrations of immunoglobulin subclasses were determined by interpolation of samples onto the reference standards.

### Fluorescent immunohistology, image capture and analysis

Serial, 5 μm sections of the ascending portion of the spiral colon and from the distal jejunum were cut using a Model OTF cryotome (Brights Instrument Company Ltd., Huntingdon. UK). Sections were air dried for 24 h then fixed by immersion in acetone for 15 min. Slides were dried prior to storage at −80 °C.

Quantification of jejunal tight cell junction (TCJ) associated proteins was carried out using: rabbit anti-mouse Zona Occludin-1 (ZO-1, Zymed, clone 61-7300) and mouse anti-rat E-cadherin (clone DECMA-1, IgG_1_), both from Serotec. Binding was detected with isotype specific antisera: goat anti-rabbit TRITC and goat anti-mouse IgG_1_-FITC (both from Southern Biotechnology, Cambridge, UK). For analysis of immunologically-relevant cells in colonic lamina propria, the monoclonal antibodies used were: anti-porcine CD16 (clone G7, IgG1); anti-porcine CD4 (clone MIL17, IgG2b); anti-porcine MIL11 (an endothelial cell-associated molecule, IgE^62^); anti-porcine MHCII DR (clone MSA3, IgG_2a_) (all from Serotec). Binding was detected with isotype specific antisera: goat anti-mouse IgG_1_-TRITC; goat anti-mouse IgG_2b_-FITC; biotinylated rat anti-mouse IgE detected with AMCA-Strepavidin (Vector Laboratories); goat anti-mouse IgG_2a_ AF633 (all Southern Biotechnology). Non-specific binding was prevented by 5% pig serum, 5% goat serum and 5% rat serum in PBS. Slides were mounted using Vectashield (Vector Laboratories). Negative control slides were prepared in conjunction with each positive slide.

Images were captured using a Leica DMR-B fluorescence microscope fitted with appropriate fluorescence filters. 10 greyscale, high quality 16-bit TIFF images were acquired using a x20 objective lens for each tissue sample (60 images/treatment group and 60 images/batch) using a Leica DC350FX camera and TWAIN-compliant software (Leica). The area of tissue staining positively with each antibody, or combination of antibodies, was quantified as we have described previously^50^ using ImageJ version 1.44^63^. Briefly, either the lamina propria (antigen presentation analysis) or epithelium (TCJ analysis) was outlined and identified as the region of interest in each image. A specifically design MACRO was used to generate a binary image of positive staining for each of the individual channels and the proportion of positive pixels for each channel, and for each of the 12 possible combinations was recorded.

### Data Analysis

For intestinal TCJ analysis following *B. lactis* NCC2818 supplementation, univariate linear regression was carried out using piglet as the experimental unit and litter, tissue and probiotic treatment as variables. Individual differences between treatment groups were determined by least-significant differences as in our previous experiments^37^. For intestinal TCJ analysis of the replicate batches, born 8 weeks apart, litter could not be used as a variable.

NMR analysis was carried out as previously described^53^. Orthogonal partial least squares discriminant analysis (O-PLS-DA) was performed in a Matlab environment using in-house algorithms^64^. To generate the serum trajectory, a series of pair-wise O-PLS-DA models were constructed at each time-point using unit variance (uv) scaling and the cross-validated scores (Tcv) were extracted for each model. The Tcv were then normalised by dividing each value by the standard deviation of the cumulative Tcv for each model, and the mean Tcv (+/− standard deviation) plotted over time. For each O-PLS-DA model showing urinary, microbial, cytokine or immunoglobulin differences, it was determined whether the Q^2^Y value (predictive ability) of the model was significantly different to the Q^2^Y value calculated from up to 2,000 random permutations of Y using the cumulative probability value determined at the 95^th^ quartile (in-house algorithm written by Dr. O.Cloarec). Models were uv scaled such that each variable had equal weight in the model, and were subject to a 7-fold cross-validation step. Correlation coefficients of >+/−0.6 (*r)* or >0.4 (*r* ^*2*^ *)*, determined by the Pearson Product-Moment coefficient as being significant at the 95% confidence interval, are highlighted in the Figures. Metabolites of interest were integrated using an in-house routine (written by R. Cavill).

Statistical analysis of 4 colour quantitative immunofluorescence was carried out using Factor Analysis in SPSS version 16 statistics (IBM SPSS inc, Chicago, US) using the proportion of pixels positive and negative for all combinations of CD4, CD16, MIL11 and MHCIIDR as variables (16 in total).

For the immuno-metabolic interactions, the NMR matrix was tested against each immunological parameter using an orthogonal partial least squares approach (O-PLS), using the NMR matrix as the X matrix and each immunological parameter as the Y matrix. Parameters that were found to be significantly associated (p < 0.05) with the NMR data were extracted and the metabolites associated with the model highlighted.

### Data availability

Data will be made available upon request.

## Electronic supplementary material

Supplementary information
