# Supplementary material for: Early intervention with Bifidobacterium lactis NCC2818 modulates the host-microbe interface independent of the sustained changes induced by the neonatal environment
Source: Sci Rep. 2017 Jul 13;7:5310. doi: 10.1038/s41598-017-05689-z (PMC5509696; doi:10.1038/s41598-017-05689-z)
Supplement: Supplementary file 1 — Supplementary information [file 41598_2017_5689_MOESM1_ESM.pdf]

Early intervention with *Bifidobacterium lactis* NCC2818 modulates the host-microbe interface independent of the sustained changes induced by the neonatal environment

Marie C. Lewis, Claire A. Merrifield, Bernard Berger, Olivier Cloarec, Swantje Duncker, Annick Mercenier, Jeremy K. Nicholson, Elaine Holmes & Mick Bailey

Table 1: Diet Composition (Calculated)

Ingredient (%)	Formula (%) (Piggimilk)	Egg-based (%)
Spray dried instant whey powder	20.0	
Whey protein concentrate (35%)	10.0	
Whey Protein concentrate (80%0	4.0	
Whole dried egg		24.3
Dairy crest tint whey	20.0	9.4
Denatured skimmed milk	43.4	7.7
Dextrose	0.8	1.7
Formula milk supplement*	1.0	
Calcium formate	0.8	
Cooked wheat (MASHM)		21.0
Presco maize		21.0
Cooked naked oats		11.7
Pig starter 210 supplement**		1.0
Dicalcium phosphate		0.9
Limestone Trical 130		0.5
L. lysine		0.4
L. threnine		0.1
Salt		0.1
Protein	24.0	21.3
Oil	18.0	12.5
Fibre	7.5	1.1
Ash	3.5	4.5
Moisture		8.6
Nitrogen free Extract NFE	47.0	52.0

***Formula milk supplement** (units in finished feed)
Vitamin A 16mg/kg; vitamin D_3 2mg/kg; vitamin E 250mg/kg; vitamin K (menadione) 4mg/kg; vitamin C 150mg/kg; plus full complement of B group vitamins.

****Piglet starter 210 supplement** (units in finished feed)
Vitamin A 16mg//kg; vitamin D_3 2mg/kg; vitamin E 250mg/kg; vitamin K (menadione) 4mg/kg; vitamin B_1 10mg/kg; vitamin B_2 16mg/kg; vitamin B_6 10mg/kg; vitamin B12 0.05mg,kg; Nicotinic acid 50mg/kg; Pantothenic acid 30mg/kg; Biotin (Vitamin K) 0.2mg/kg; Vitamin C 200mg/kg; Folic acid 3mg/kg; Choline Chloride 300mg/kg.
Trace minerals: Copper 155mg/kg; Iron 375mg/kg; Zinc 110mg/kg, Manganese 100mg/kg; Cobalt 0.5mg/kg; Iodine 1.2mg/kg; Selenium 0.3mg/kg.

Supplementary Table 1. Composition of formula milk and egg-based weaning diet

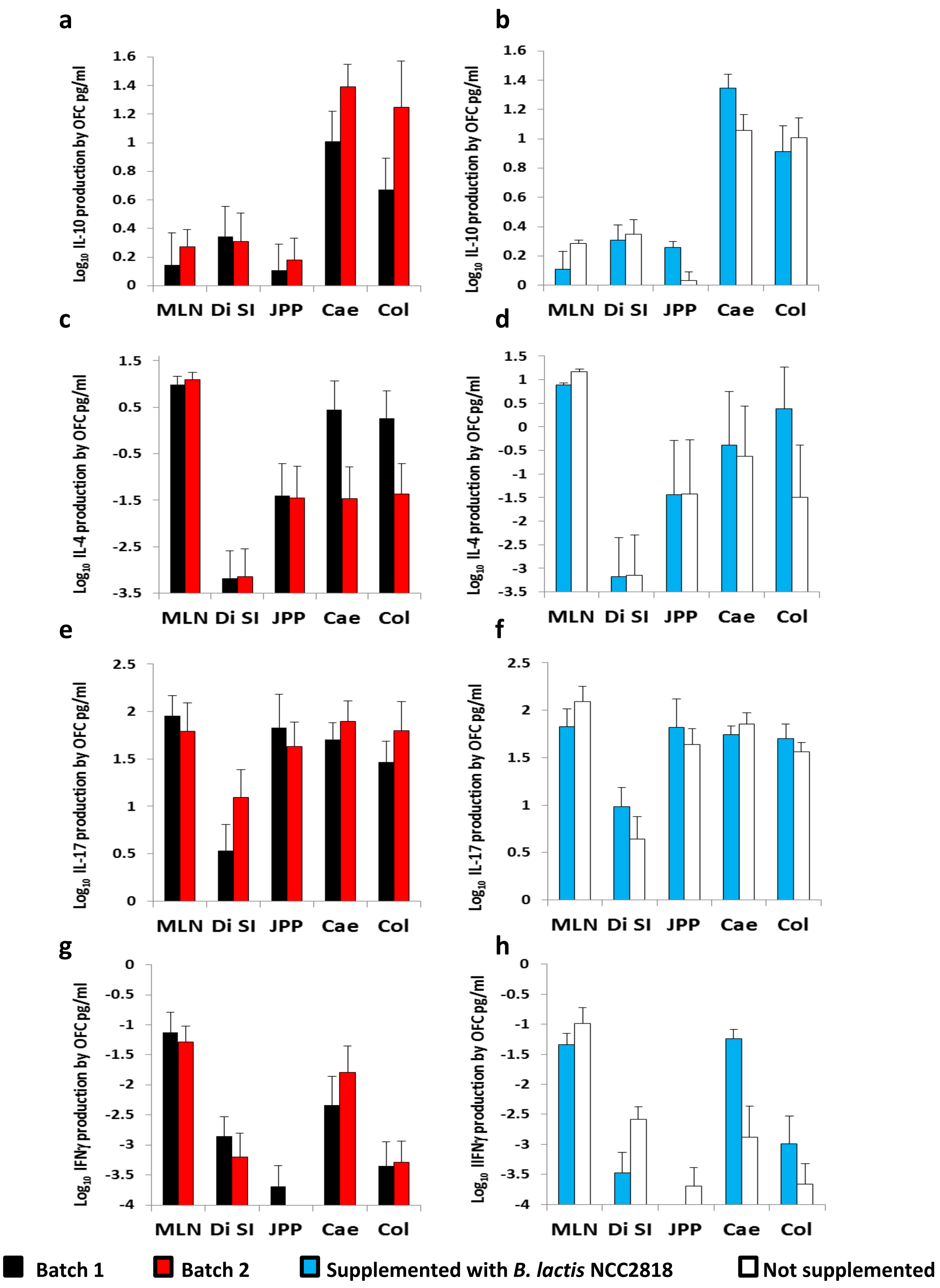

Supplementary Figure 1: Cytokine production by organ fragment cultures (OFC) of the intestinal-associated tissues mesenteric lymph node (MLN), distal small intestine (Di SI), jejunal Peyer's patch (JPP), caecum (Cae) and colon (Col) by batch (left column. black, batch 1; red, batch 2) and by treatment (right column. supplemented with *B. lactis* NCC2818, blue; unsupplemented control, white). Where **a** and **b** are IL-10, **c** and **d** are IL-4, **e** and **f** are IL-17 and **g** and **h** are IFNγ (Error bars=SEM; n=6).

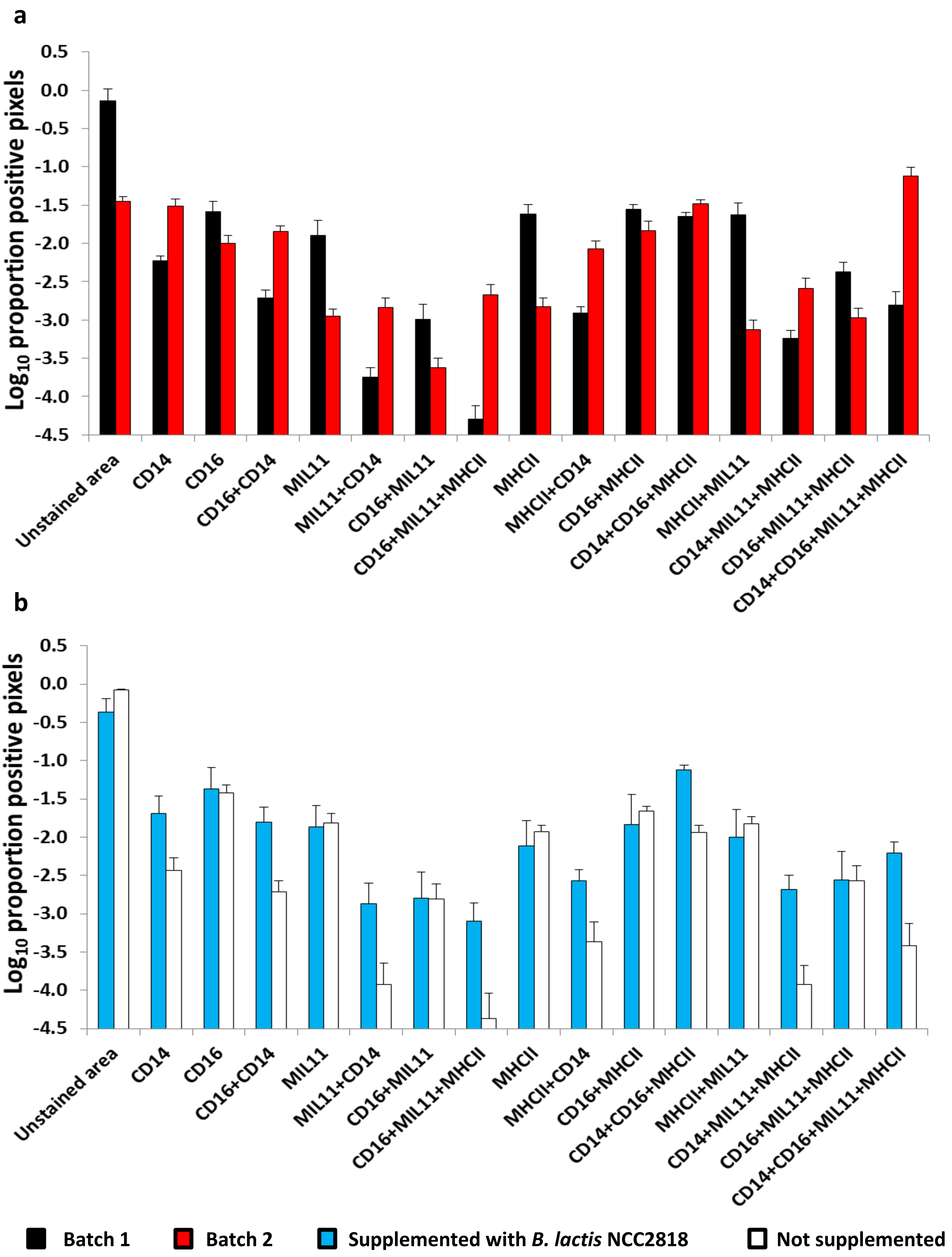

Supplementary Figure 2. Log_{10} of the proportion of colonic tissue staining positive for CD14, CD16, capillary endothelium (MIL11) and MHCIIDR (and all combinations) was quantified using 4-colour fluorescence immunohistology by batch (**a**) and by treatment (**b**) where batch 1, black; batch 2, red; supplementation with *B. lactis* from 2 days onwards, blue; control (no supplementation, white. (Error bars=SEM; n=6).

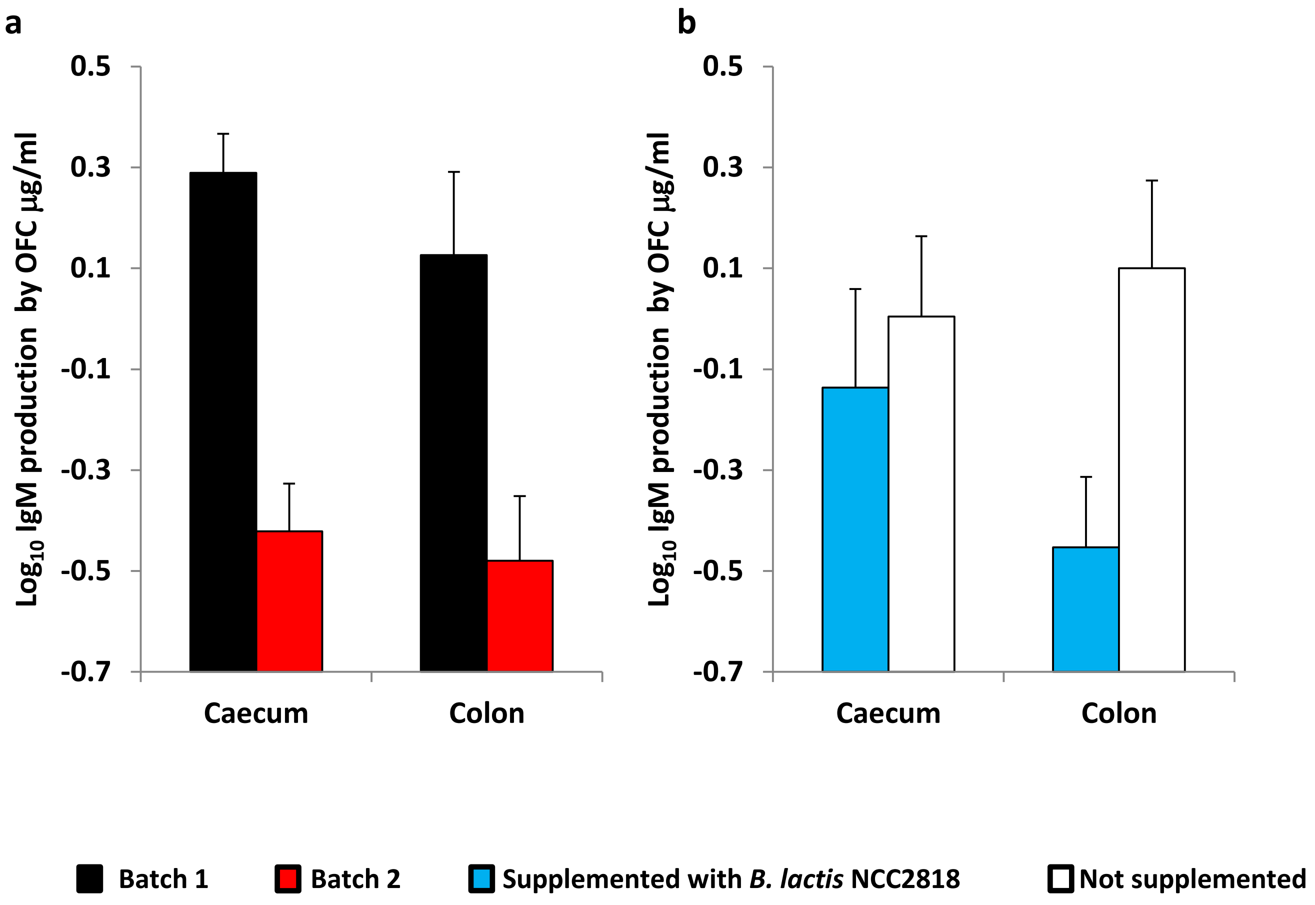

Supplementary Figure 3. Immunoglobulin M (IgM) production by organ fragment cultures (OFC) of caecum (Cae) and colon (Col) by batch (**a** black, batch 1; red, batch 2) and by treatment (**b** supplemented with *B. lactis* NCC2818, blue; unsupplemented control, white). (Error bars=SEM; n=6).

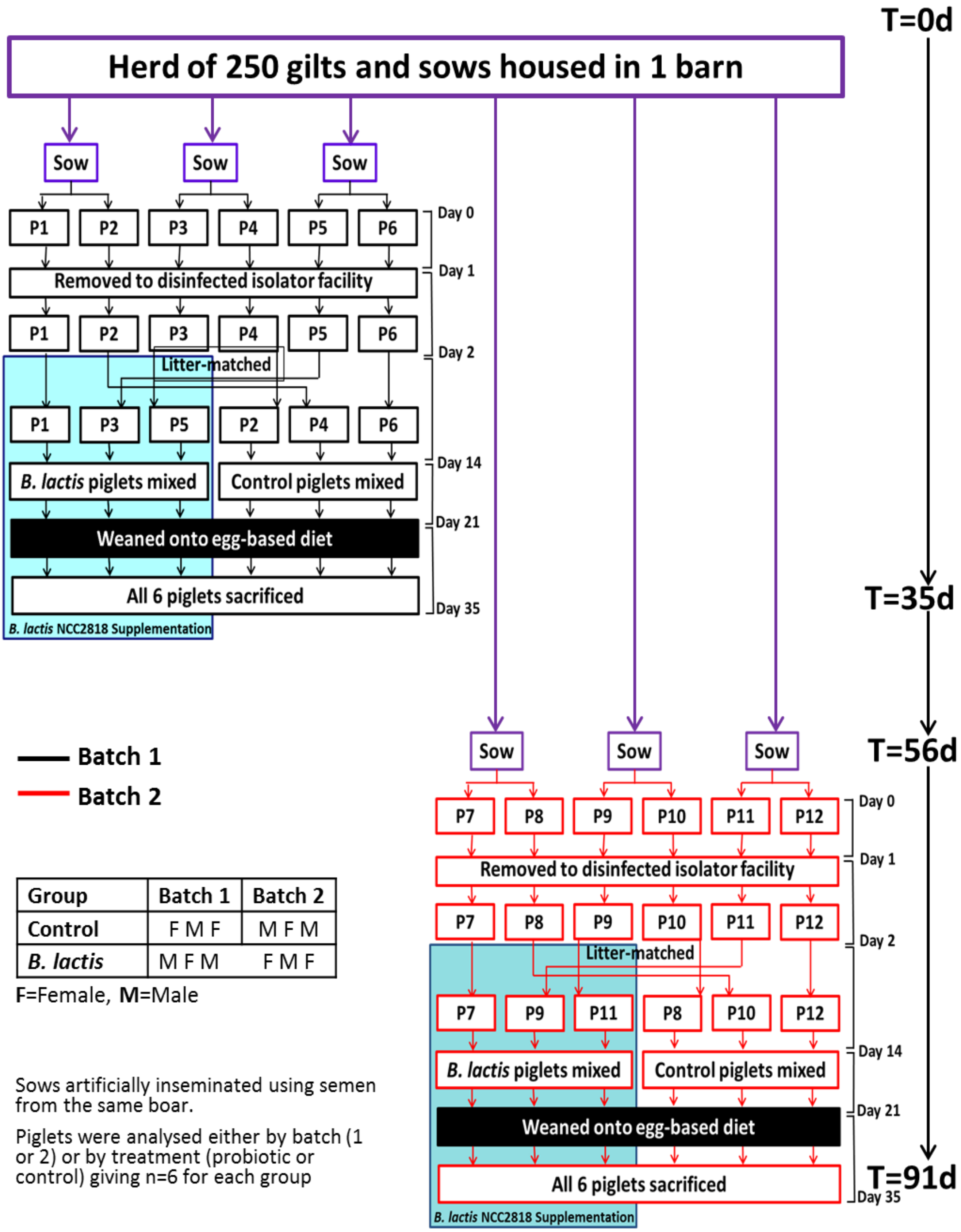

Group	Batch 1	Batch 2
Control	F M F	M F M
B. lactis	M F M	F M F

F=Female, **M**=Male

Sows artificially inseminated using semen from the same boar.

Piglets were analysed either by batch (1 or 2) or by treatment (probiotic or control) giving n=6 for each group

Supplementary Figure 4: Detailed schematic of experimental design
